# Supplementary material for: Depletion of Paraoxonase 1 (Pon1) Dysregulates mTOR, Autophagy, and Accelerates Amyloid Beta Accumulation in Mice
Source: Cells. 2023 Feb 26;12(5):746. doi: 10.3390/cells12050746 (PMC10001133; doi:10.3390/cells12050746)
Supplement: Supplementary file 1 [file cells-12-00746-s001.zip › cells-2204117-supplementary.pdf]

Depletion of paraoxonase 1 (Pon1) dysregulates mTOR, autophagy, and accelerates amyloid beta accumulation in mice

Łukasz Witucki and Hieronim Jakubowski

Supplementary Material
Supplementary **Figure S1**
Supplementary **Figure S2**
Supplementary **Table S1**

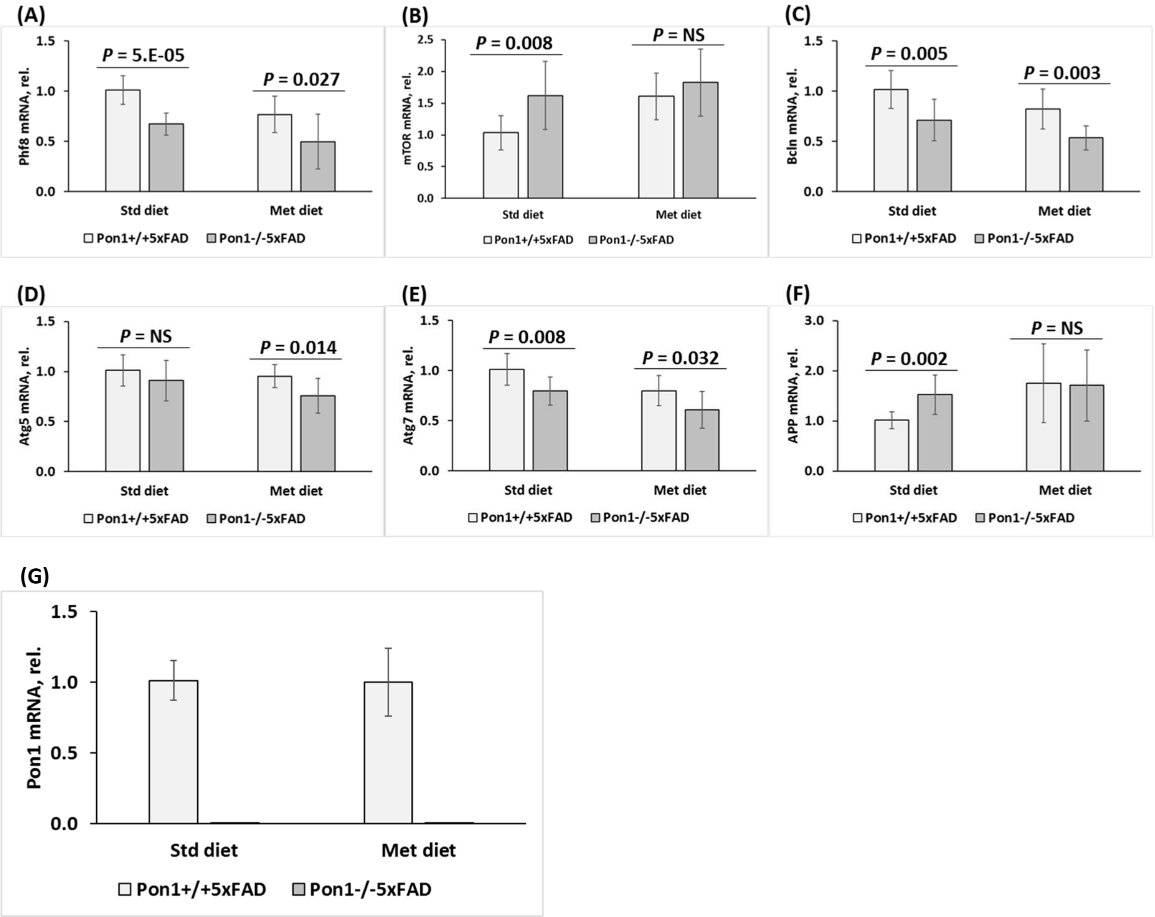

Figure S1. Pon1 depletion affects the expression of mRNAs for Phf8, mTOR, autophagy-related proteins, and App in the *Pon1*$^{-/-}$5xFAD mouse brain. Bar graphs illustrating the quantification by RT-qPCR of mRNAs for Phf8 (**A**), mTOR (**B**), Bcln1 (**C**), Atg5 (**D**), Atg7 (**E**), and App (**F**) are shown. Pon1 mRNA was absent in *Pon1*$^{-/-}$5xFAD brains (**G**). Met diet did not affect Pon1 mRNA in *Pon1*$^{+/+}$5xFAD mouse brain. Gapdh mRNA was used as a reference.

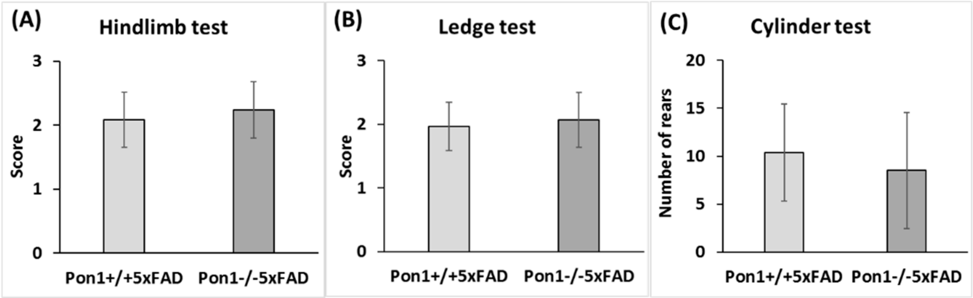

Figure S2. Pon1 depletion did not affect sensorimotor activity in mice. Behavioral performance of 12-month-old *Pon1*$^{-/-}$ 5xFAD (n = 21) and *Pon1*$^{+/+}$5xFAD (n = 18) mice was assessed in the hindlimb clasping test (**A**), ledge test (**B**), and cylinder test (**C**).

Table S1 Primers used for PCR or RT-qPCR

**Gene**	**Primer sequence**
APP	*Forward: 5'-CTTCCCCAAGATCCTGATAAACT-3'*
	Reverse: 5'-CCGGGTGTCTCCAGGTACT-3'
Atg5	*Forward: 5'-AAGGCACACCCCTGAAATGG-3'*
	Reverse: 5'-TGATGTTCCAAGGAAGAGCTGAA-3'
Atg7	*Forward: 5'-GCCAACTCCACACTGCTTTC-3'*
	Reverse: 5'-TCTTCTGGGTCAGTTCGTGC-3'
Beclin-1	*Forward: 5'GAG GAA GCT CAG TAC CAG CG 3'*
	Reverse: 5'CCA GAT GTG GAA GGT GGC AT 3'
Pon1	*Forward p1: 5'-CACTGTAGCTGTACTCACAC-3'*
	Reverse p2: 5'-ATAGGAAGACCGATGGTTCT-3';<br>*Reverse p3 (5'-TCCTCGTGCTTTACGGTATCG-3')*<br>*(neomycin cassette)*
Gapdh	*Forward: 5'-GGACTGGATAAGCAGGGCG-3'*
	Reverse: 5'-TTTTGTCTACGGGACGAGGC-3'
mTOR	*Forward: 5'-GCCACTCTCTGACCCAGTTC-3'*
	Reverse: 5'-ATGCCAAGACACAGTAGCGG-3'
Phf8	*Forward: 5'-TGGGAGCATGCTTCAAGG-3'*
	Reverse: 5'-GATTTCAAAGCAGGGTCATCA-3'
hAPP transgene in 5xFAD mice	*Forward: 5'-AGAGTACCAACTTGCATGACTACG-3';*
	Reverse: 5'-ATGCTGGATAACTGCCTTCTTATC-3'
hPS1 transgene in 5xFAD mice	*Forward: 5'-GCTTTTTCCAGCTCTCATTTACTC-3'*
	Reverse: 5'-AAAATTGATGGAATGCTAATTGGT-3'
*mTOR upstream TSS**	*Forward: 5'-TTGCCAACTGGTGCTCGTTT-3'*
	Reverse: 5'-AAGAATTGGAGCTCGGGACC-3'
*mTOR TSS**	*Forward: 5'-GGATGTTCCTCCCCAATCTTCG-3'*
	Reverse: 5'-CAGACCCACCTAACTGACCGT-3'
*mTOR downstream TSS**	*Forward: 5'-TAGGGGGCAGATCCCGAAAC-3'*
	Reverse: 5'-CACTGTAGCTGTAACTCACAC-3'
** TSS, transcriprtion start site*	
